# Supplementary material for: Bayesian Spatio-Temporal Multilevel Modelling of Patient-Reported Quality of Life following Prostate Cancer Surgery
Source: Healthcare (Basel). 2024 May 26;12(11):1093. doi: 10.3390/healthcare12111093 (PMC11171974; doi:10.3390/healthcare12111093)
Supplement: Supplementary file 1 [file healthcare-12-01093-s001.zip › Supplementary file S1.pdf]

Supplementary file S1

Description of Bayesian spatial-temporal multilevel model

Individual level outcome model

Individual level covariates and outcome are obtained from PCOR-Vic registry from 2015 to 2021. We have a binary outcome Y_{ijt} from a sample of individuals i (i=1,2,3….5238) from a set of area j(j=1,2,3……..79) and time year (t=1,2,3 …..7). The binary outcome Y_{ijt} follows a Bernoulli distribution as

$$Y_{ijt} \sim Bernoulli(p_{ijt}) \text{ ……………………………………………………… (1)}$$

where i(individual), j(area) and t(year)

$$logit(p_{ijt}) = \mu + \beta X_{ijt} + \gamma Z_j + v_j + u_j + t_t \text{ …………………………………… (2)}$$

Aggregate data

Area level variables like Index of Relative Socio-economic Disadvantage (IRSD) and remoteness were sourced from the Australian Bureau of Statistics (ABS)(1). Let Y_{jt} area level outcome with N_{jt} population and $\boldsymbol{P_{jt}}$ probability has the following distribution.

$$Y_{jt} \sim binomial(N_{jt}, P_{jt}) \text{ ……………………………………………………… (3)}$$

$$\boldsymbol{P}_{jt} = \sum_k f_{jtk}\, P_{jtk} \text{ ………………………………………………………… (4)}$$

$$P_{jt} = \sum_{r1,r2,r3,r4,r5,r6,r7} f_{\{j,t,r1,r2,r3,r4,r5,r6,r7\}}\, p_{\{j,t,r1,r2,r3,r4,r5,r6,r7\}} \text{ …………………… (5)}$$

Where f_{jtk} is within area probability distribution of individual covariates, P_{jtk} is outcome probability of individual in area j and category k, where k is risk factor combination i.e r1*r2*…. *r7.

$$logit(p_{\{j,t,r1,r2,r3,r4,r5,r6,r7\}}) = \mu + \beta X_{jtk} + \gamma Z_j + v_j + u_j + t_t + t_t^2 \text{ ……………… (6)}$$

Where v_j is spatially structured random effect, u_j is spatially unstructured random effect, t_t is linear time and t_t^2 quadratic trend time.

The Bayesian framework was employed to fit the model, utilizing weakly informative priors. This approach serves to effectively regularize sparse data settings within the Hierarchical Risk and Reliability (HRR) methodology(2, 3). We specified priors for fixed-effects individual-level covariates and random effect area-level covariates, setting their means to zero and precision to

0.725. This was done under the assumption of a 95% probability that the true odds ratio (OR) falls within the range of 0.1 to 10.

Weight matrices specification

In the development of models, the selection of a spatial weight matrix plays a crucial role in both model fitting and parameter estimation. Various methods exist for specifying spatial weight matrices. The most common methods are adjacency based and distance-based weight matrices(4, 5). The contiguity or adjacency-based weight matrices expressed as mathematically, spatial structured random effect u_i represented as

$$\left(u_i \middle| u_j,\ \ i \neq j\right) \sim N(\overline{u_\iota}, \tau_i^2) \ldots\ldots\ldots\ldots\ldots\ldots\ldots\ldots\ldots\ldots\ldots\ldots (7)$$

Where $\overline{u_\iota} = \frac{1}{\sum_j wij} \sum ujwij$

$$\tau_i^2 = \frac{\tau_u^2}{\sum_j wij}$$

$$w_{ij} = \begin{cases} 1 & if\ area\ i\ and\ j\ are\ \ neighbours \\ 0 & otherwise \end{cases} \ldots\ldots\ldots\ldots\ldots\ldots\ldots\ldots\ldots (8)$$

A frequently used mathematical representation for distance-based neighbours involves expressing their relationship as a function of geographic distance. The distance between area i and area j is computed as the Euclidean distance between their respective centroids.

$$\mathrm{w_{ij}} = \left(\frac{1}{d_{ij}}\right)^k \ldots\ldots\ldots\ldots\ldots\ldots\ldots\ldots\ldots\ldots\ldots\ldots\ldots\ldots\ldots\ldots\ldots\ldots\ldots (9)$$

For a positive integer k, commonly set to 1, the impact of neighbouring areas increases as the exponent k grows larger, emphasizing the influence of nearby regions over those that are more distant. The w_{ij} take 1 and 0 as follows.

$$w_{ij} = \begin{cases} 1 & if\ area\ i\ and\ j\ are\ \ neighbours\ based\ on\ given\ distance\ band \\ 0 & otherwise \end{cases} \ldots\ldots\ldots\ldots\ldots\ldots (10)$$

Relative contribution of structured and unstructured variations in model using distance based and adjacency-based weight matrices.

The relative contribution of spatial variation was calculated by dividing variance of structured random effect var(u) by sum of variance of structure var(u) and variance of unstructured random effect var(v) at postcode and LGA level, empirically represented as

$$\emptyset = \frac{\boldsymbol{var(u)}}{\boldsymbol{var(u)+var(v)}} \quad \ldots\ldots\ldots\ldots\ldots\ldots\ldots\ldots \textbf{(11)}$$

If value of ∅ is closed to 1 means that spatially structured random variation effect dominates while value of ∅ is closed to 0 means that unstructured random effect variation dominates.

Reference

1. Statistics ABO. Census of population and housing. Australian Government, Canberra. 2006.
2. Jackson C, Best, Nicky, Richardson S. Hierarchical related regression for combining aggregate and individual data in studies of socio-economic disease risk factors. Journal of the Royal Statistical Society Series A: Statistics in Society. 2008;171(1):159-78.
3. Jonker MF, Donkers B, Chaix B, van Lenthe FJ, Burdorf A, Mackenbach JP. Estimating the impact of health-related behaviors on geographic variation in cardiovascular mortality: a new approach based on the synthesis of ecological and individual-level data. Epidemiology. 2015;26(6):888-97.
4. Duncan EW, White NM, Mengersen K. Spatial smoothing in Bayesian models: a comparison of weights matrix specifications and their impact on inference. International journal of health geographics. 2017;16(1):1-16.
5. Earnest A, Morgan G, Mengersen K, Ryan L, Summerhayes R, Beard J. Evaluating the effect of neighbourhood weight matrices on smoothing properties of Conditional Autoregressive (CAR) models. International journal of health geographics. 2007;6(1):1-12.
