# Supplementary material for: Bayesian Spatio-Temporal Multilevel Modelling of Patient-Reported Quality of Life following Prostate Cancer Surgery
Source: Healthcare (Basel). 2024 May 26;12(11):1093. doi: 10.3390/healthcare12111093 (PMC11171974; doi:10.3390/healthcare12111093)
Supplement: Supplementary file 1 [file healthcare-12-01093-s001.zip › Supplementary file S2.pdf]

Neighbourhood type	Histogram	Connectivity map	Connectivity graph
Distance based weight matrix	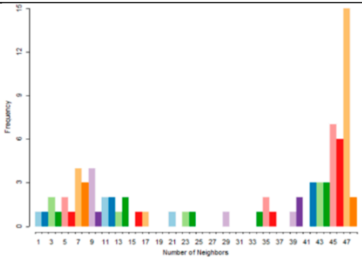	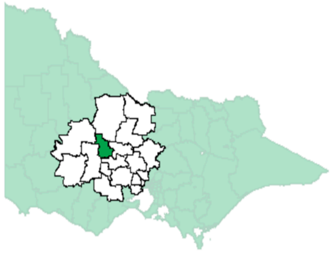	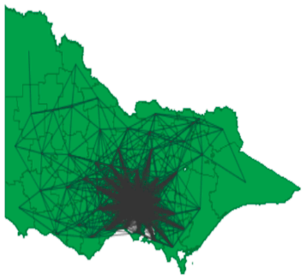
Adjacency based weight matrix (Queens-I)	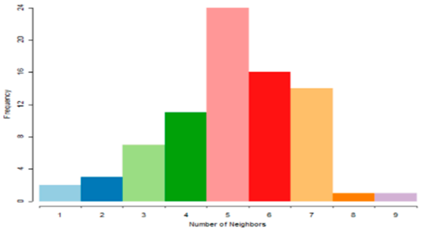	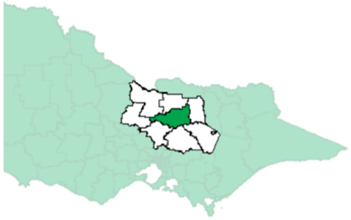	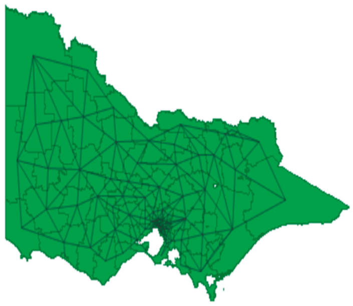

Supplementary file S2: Histogram, connectivity map and connectivity graph for distance based and adjacency-based weight matrices.
