# Supplementary figures and images for: Bayesian Spatio-Temporal Multilevel Modelling of Patient-Reported Quality of Life following Prostate Cancer Surgery
Source: Healthcare (Basel). 2024 May 26;12(11):1093. doi: 10.3390/healthcare12111093 (PMC11171974; doi:10.3390/healthcare12111093)

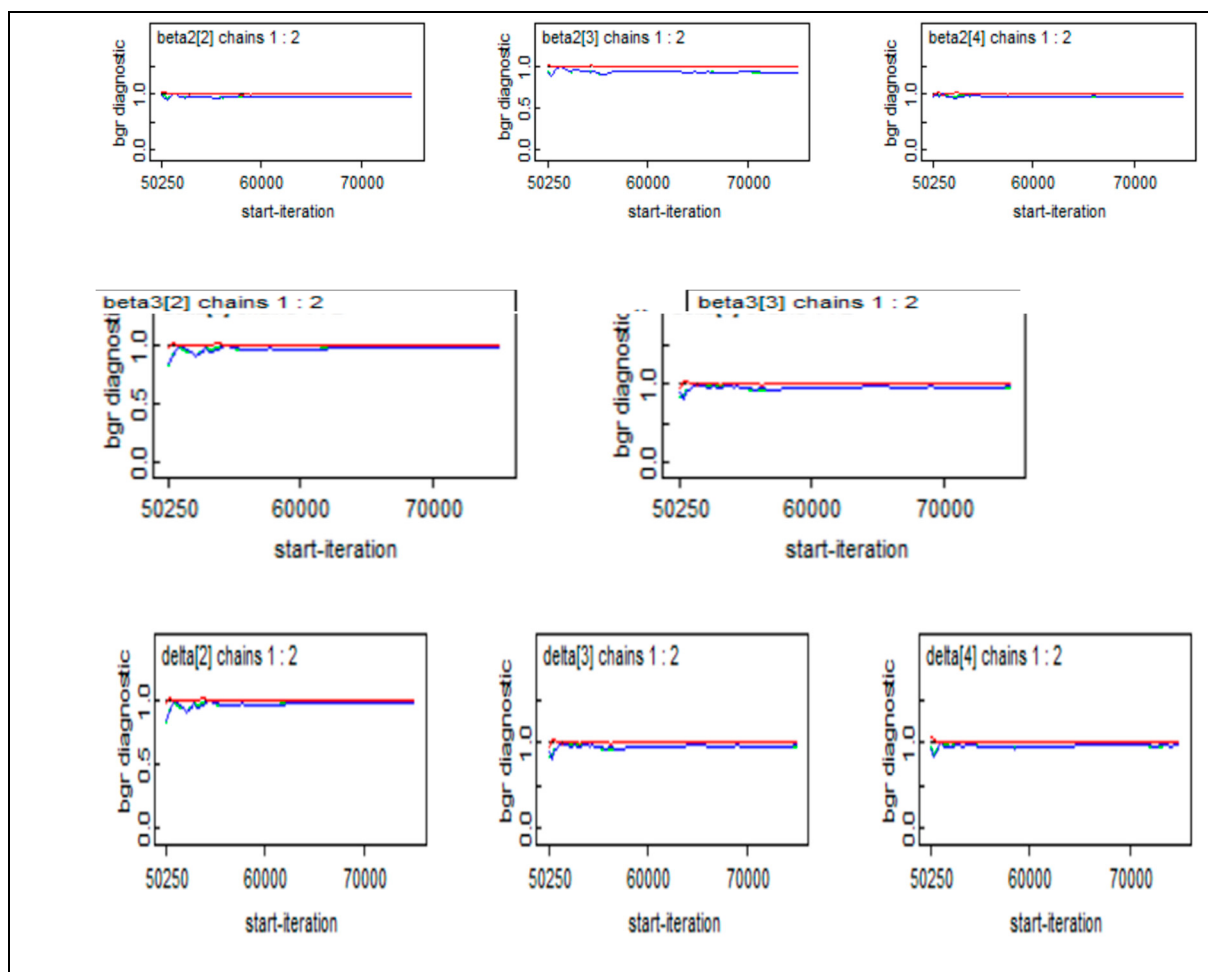

Supplementary file S3: - Gelman Rubin statistics plots for assessing model convergence.

Supplement: Supplementary file 1 [file healthcare-12-01093-s001.zip › Supplementary file S3.pdf]
